# Polycomb protein RYBP activates transcription factor *Plagl1* during *in vitro* cardiac differentiation of mouse embryonic stem cells

**DOI:** 10.1098/rsob.220305

**Published:** 2023-02-08

**Authors:** Surya Henry, Lilla Kokity, Melinda Katalin Pirity

**Affiliations:** ^1^ Institute of Genetics, Biological Research Centre, Eötvös Loránd Research Network, 6726 Szeged, Hungary; ^2^ Doctoral School in Biology, Faculty of Science and Informatics, University of Szeged, 6726 Szeged, Hungary

**Keywords:** stem cell, *in vitro* cardiac differentiation, RYBP, transcriptional regulation, *Plagl1*

## Abstract

RING1 and YY1 binding protein (RYBP) is primarily known to function as a repressor being a core component of the non-canonical polycomb repressive complexes 1 (ncPRC1s). However, several ncPRC1-independent functions of RYBP have also been described. We previously reported that RYBP is essential for mouse embryonic development and that *Rybp* null mutant embryonic stem cells cannot form contractile cardiomyocytes (CMCs) *in vitro*. We also showed that PLAGL1, a cardiac transcription factor, which is often mutated in congenital heart diseases (CHDs), is not expressed in *Rybp*-null mutant CMCs. However, the underlying mechanism of how RYBP regulates *Plagl1* expression was not revealed. Here, we demonstrate that RYBP cooperated with NKX2-5 to transcriptionally activate the *P1* and *P3* promoters of the *Plagl1* gene and that this activation is ncPRC1-independent. We also show that two non-coding RNAs residing in the *Plagl1* locus can also regulate the *Plagl1* promoters. Finally, PLAGL1 was able to activate *Tnnt2*, a gene important for contractility of CMCs in transfected HEK293 cells. Our study shows that the activation of *Plagl1* by RYBP is important for sarcomere development and contractility, and suggests that RYBP, via its regulatory functions, may contribute to the development of CHDs.

## Graphical abstract

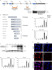

## 1. Introduction

Polycomb proteins (PcGs) are epigenetic regulators with distinct functions in maintaining cell identity during mouse embryonic development [1]. PcGs physically associate with form polycomb repressive complexes (PRCs) [2]. Biochemical analysis of PRC complexes revealed their diverse compositions; based on their association they were classified accordingly as the canonical PRC1 (cPRC1), the non-canonical PRC1 (ncPRC1) and the PRC2 complex [3]. Homozygous null mutations of the PRC complex members resulted in the upregulation of many genes in the embryonic stem (ES) cells indicating their role as repressors [4,5]. Although few genes are always downregulated in the homozygous PcG null mutants, we have no clear understanding about how PRCs can perhaps activate gene targets [6,7].

RING1 and YY1 binding protein (RYBP, also known as death effector domain [DED]-associated factor DEDAF) is a core member of the ncPRC1 complexes and is classically highlighted for its role as a repressor [5,8,9]. RYBP is also described as a protein with the ability to interact with multiple partners playing roles in diverse biological functions [10]. Previous publications from our and other laboratories have demonstrated the essential role of RYBP in early mouse embryonic development affecting the formation of the central nervous system (CNS), hematopoietic system and the eye [11–13]. Previously, we have also reported that ES cells lacking RYBP could not form functionally contracting cardiomyocytes (CMCs) *in vitro.* While the precise mechanism of this phenotype has not been revealed yet, we have earlier shown that CMCs exhibited impairment in their ion homeostasis, cell adhesion, cardiac progenitor and sarcomere formation as key molecular mechanisms that contributed to the non-contractility phenotype of the *Rybp^-/-^* CMCs [14]. Strikingly, pleiomorphic adenoma gene-like 1 (*Plagl1*, also called as zinc finger protein regulator of apoptosis and cell cycle arrest (*Zac1*)) was absent in the *Rybp^-/-^* ES cells and differentiated CMCs as identified by transcriptome analysis [15]. PLAGL1 is a cardiac transcription factor (TF) shown to be expressed in a chamber-restricted manner in the developing mouse embryonic heart [16]. Impaired expression of *Plagl1* is implicated in the formation of congenital heart diseases (CHDs) such as the atrial septum defect [16]. Recently, aberrations in the imprinting of the *Plagl1* locus were directly connected to the formation of CHDs [17].

In this study, we used mouse ES cells to explore the role of *Plagl1* during CMC development *in vitro* and discovered a mechanism of RYBP action regulating the expression of *Plagl1.* Here we found that *Plagl1* expression increased from the progenitor formation stages and expressed prominently during the late stages of *in vitro* cardiac differentiation. We present evidence that RYBP activated the expression of *Plagl1* by associating with cardiac TF NK2 homeobox 5 (NKX2-5). We have also demonstrated that the two non-coding RNAs (ncRNAs) hydatidiform mole-associated and imprinted (*Hymai*) and plagl1 intronic transcripts (*Plagl1it*), which reside in the *Plagl1* genomic locus, may also participate in the regulation of the *Plagl1* promoters. Our results highlight a ncPRC1-independent role of RYBP in the regulation of the *Plagl1* gene expression and also provide examples of an activator role of RYBP during development.

## 2. Results

### 2.1. The expression of Plagl1, Hymai and Plagl1it is severely downregulated in the Rybp^-/-^ cardiomyocytes

**Figure 1.**
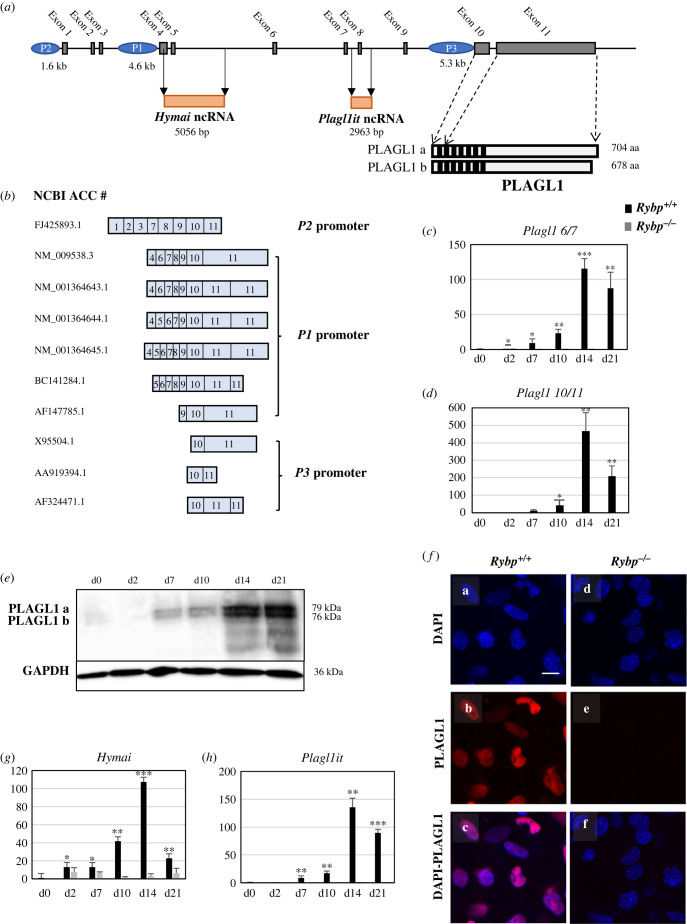
*Plagl1* is not present in the *Rybp*^-/-^ cells during *in vitro* cardiac differentiation of mouse ES cells*.* (*a*) Schematic representation of the *Plagl1* genomic locus. Exons are represented with grey bars; the three promoters *P1*, *P2* and *P3* are marked in blue ovals; the two ncRNAs, *Hymai* and *Plagl1it* are represented with orange rectangles. (*b*) Schematic representation of the various splice variants of *Plagl1*. NCBI accession numbers are presented on the left and corresponding promoters are at the right of different splice variants. (*c*,*d*) Relative gene expression analysis of *Plagl1* using primers specific to (*c*) exon 6/7 and (*d*) exon 10/11 during *in vitro* cardiac differentiation by qRT-PCR analysis. (*e*) Western blot analysis of PLAGL1 during *in vitro* cardiac differentiation. GAPDH was used as internal loading control. (*f*) ICC analysis of d14 *in vitro* cardiac differentiated samples stained with PLAGL1. Relative gene expression analysis of (*g*) *Hymai* and (*h*) *Plagl1it* ncRNA during cardiac differentiation. Immunostaining: blue: DAPI (nuclei); red: PLAGL1. Olympus Confocal IX 81, obj.: 60 x; Scale bar in (*a*–*f*): 20 µm. Abbreviations: d, day.

To investigate the possible mechanism of how RYBP can regulate *Plagl1* expression during *in vitro* cardiac differentiation, we first studied the structure of the *Plagl1* genomic locus [18]. *Plagl1* has a complex genomic structure containing 11 exons, 3 promoter regions *P1*, *P2* and *P3* and 2ncRNAs (figure 1*a*) [19]. The *Plagl1 P1* promoter contains a 1 kb long CpG island and is part of a differentially methylated region (DMR) which serves as the site of imprinting for the genomic locus. There are also two ncRNAs, *Hymai* and *Plagl1it*, located downstream to the DMR. Previous studies have identified biallelic expression of *Plagl1* from an alternate promoter *P2* situated 30 kb upstream to the *P1* promoter (site of imprinting) in patients with transient neonatal diabetic mellitus (TNDM) [20]. Later studies have also identified the presence of a novel alternate *P3* promoter which lies immediately upstream to the start codon in exon 10 of the *Plagl1* locus [21]. *Hymai* shares its promoter with *Plagl1* at the *P1* promoter while there is no clear information about the promoter/enhancer region corresponding to *Plagl1it*. In order to understand which promoter can produce protein-coding transcripts and which promoters are active during *in vitro* cardiac differentiation we analysed available expressed sequence tag (EST) data. From the deposited *Plagl1* transcripts in EST database as shown in figure 1*b*, only FJ425893.1 is transcribed from the *P2* promoter, NM_009538.3, NM_001364643.1, NM_001364644.1, NM_001364645.1, BC141284.1 and AF147785.1 are transcribed from the *P1* promoter and X95504.1, AA919394.1 and AF324471.1 are transcribed from the *P3* promoter. This suggested that all three promoters can produce mRNA transcripts; however, this analysis did not give any information about the promoters active during cardiac differentiation. To gain insights about the expression kinetics of *Plagl1* through the time course of *in vitro* cardiac differentiation, wild-type (*Rybp^+/+^*) and *Rybp* null mutant ES cells (*Rybp^-/-^*) were differentiated to form CMCs for up to 21 days, as we previously reported [15]. In brief, ES cells were let to form embryoid bodies (EBs) for 2 days upon withdrawal of leukaemia inhibitory factor (LIF), a factor essential for maintaining pluripotency in mouse ES cells. On the second day, the EBs were harvested and seeded on gelatine-coated dishes and cultured for up to 21 days. Whole-cell RNA was isolated from d0 (pluripotent stem cell stage), d2 (EBs stage), d7 (cardiac progenitor formation stage), day 10 (late cardiac progenitor formation stage), d14 and d21 (terminal cardiac stage) and used for gene expression analysis by quantitative real-time PCR (qRT-PCR) (see Material and methods) (electronic supplementary material, figure S1a). We investigated the *Plagl1* expression kinetics using primers specific to the exons that are distinctive to the transcripts produced from each promoter (i.e. *P1*, *P2* and *P3*). We used primers specific to exon 1 and 2 (hereafter mentioned as *Plagl1 1/2*) to check the expression from the *Plagl1 P2* promoter, primers specific to exon 6 and 7 (hereafter mentioned as *Plagl1 6/7*) to check the expression from the *Plagl1 P1* promoter. We used primers specific to exon 10 and 11 as a universal primer pair to detect all the splice variants of *Plagl1*. QRT-PCR analysis using *Plagl1 6/7* primers in the wild-type cultures revealed weak *Plagl1* expression until d7 and its expression levels induced to over 100 folds by d14 (figure 1*c*)*.* Using the *Plagl1* 10/11 primers, the expression level of *Plagl1* was induced to over 400-fold in d14 (figure 1*d*) when compared to the 100-fold induction in *Plagl1 6/7* suggesting that both *Plagl1 P1 and P3* promoters could be presumably active during *in vitro* cardiac differentiation (figure 1*c*,*d*). Using primers specific to *Plagl1 1/2*, we did not get any product in the wild-type cells (electronic supplementary material, figure S1*b*), suggesting that the *P2* promoter may not be active during cardiac differentiation as expected. In the *Rybp^-/-^* cells, we could not detect any *Plagl1* transcripts from any of the three promoters, as expected.

To determine if the PLAGL1 protein (PLAGL1) can be detected at any stage of *in vitro* cardiac differentiation in the *Rybp* null mutant CMCs, we performed Western blot analysis using whole cell lysates derived from designated time points of *in vitro* cardiac differentiation (i.e. d0, d2, d7, d10, d14 and d21) (see Material and methods) (electronic supplementary material, figure S1*a*). Western blot was performed by hybridizing the membranes with anti-PLAGL1 antibody, and GAPDH was used as an internal loading control (figure 1*e*). In the wild-type cultures, bands corresponding to the two *Plagl1* isoforms (PLAGL1 a: 79 kDa and PLAGL1 b: 76 kDa) were detected from d7. PLAGL1 level was weak until d7 and expressed abundantly in the terminal stages of cardiac differentiation (i.e. d14 and d21) (figure 1*e*) correlating to its mRNA expression (figure 1*c*,*d*; electronic supplementary material, figure S1*c*). PLAGL1 was not detectable at any examined time points of cardiac differentiation in the *Rybp* null mutant cultures (figure 1*f*). Immunocytochemical (ICC) analysis using d14 CMCs from both wild-type and *Rybp* null mutant cultures confirmed that PLAGL1 signal was strongly detected in the wild-type d14 *in vitro* cardiac differentiated cells (PLAGL1, figure 1*f*(b,c)), and PLAGL1 signal was not observed in the *Rybp^-/-^* cultures (figure 1*f*(e,f); electronic supplementary material, figure S1C) by staining samples with anti-PLAGL1 antibody (see Material and methods).

Next, we investigated whether the expression of the two ncRNAs in the *Plagl1* locus (*Hymai* and *Plagl1it*) were also affected in the *Rybp* null mutant cells in comparison to the wild-type. QRT-PCR analysis showed that the expression of both *Hymai* and *Plagl1it* was similar to the expression kinetics of *Plagl1* in the wild-type CMCs*. Hymai* and *Plagl1it* expressed weakly until d7 and their expression gradually increased as differentiation proceeded (figure 1*g*,*h*). At d14, both *Hymai* and *Plagl1it* expression peaked up to 100-fold compared to d0 implying that the two ncRNAs may also have some roles during *in vitro* cardiac differentiation (figure 1*g*,*h*).

Results above demonstrated that both at the mRNA and protein level there is impairment in the output of the genomic products from the *Plagl1* locus in the *Rybp* null mutant cells suggesting a possible regulatory role of RYBP at the *Plagl1* locus during *in vitro* cardiac differentiation.

### 2.2. RYBP and PLAGL1 are co-localized in the differentiating cardiomyocytes and the expression of Plagl1 is induced from the cardiac progenitor formation stages

**Figure 2.**
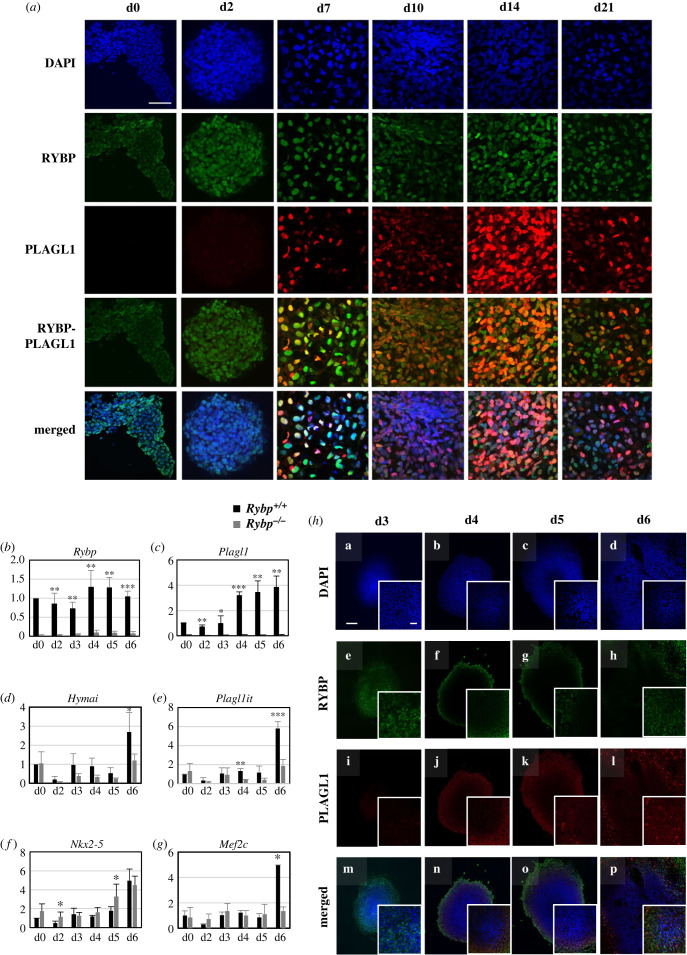
RYBP and PLAGL1 are co-expressed in the differentiating wild-type cardiac cultures. (*a*) ICC analysis for the subcellular localization of RYBP and PLAGL1 in wild-type cultures from d0, d2, d7, d10, d14 and d21 time points of *in vitro* cardiac differentiation. Time points of sample collection are shown on top of the image. Immunostainings: blue: DAPI (nuclei); green: RYBP; red: PLAGL1. Olympus Confocal IX 81, obj: 60×. Scale bar: 100 µm. (*b*–*g*) Relative gene expression analysis of (*b*) *Rybp,* (*c*) *Plagl1*, (*d*) *Hymai*, (*e*) *Plagl1it*, (*f*) *Nkx2-5* and (*g*) *Mef2c* by qRT-PCR in samples derived from *in vitro* cardiac differentiation at d0, d2, d3, d4, d5 and d6. The results represent the mean ± s.e.m. of three independent experiments. Values of *p* < 0.05 were accepted as significant (* *p* < 0.05; ** *p* < 0.01; *** *p* < 0.001). Statistical method: *t*-test type 3. (*h*) ICC analysis of RYBP and PLAGL1 at d0, d2, d3, d4, d5 and d6 samples of *in vitro* cardiac differentiated samples. Immunostainings: blue: DAPI (nuclei); green: RYBP; red: PLAGL1. Olympus Confocal IX 81, obj: 20×. Scale bar: 100 µm.

In order to compare the subcellular localization of RYBP and PLAGL1, we performed ICC analysis on samples of cardiac differentiation of wild-type ES cells (i.e. d0, d2, d7, d10, d14 and d21). Differentiating CMCs were cultured on glass coverslips, fixed with 4% PFA and mounted on glass slides (see Material and methods). The samples were co-stained with anti-RYBP and anti-PLAGL1 antibodies. In the pluripotent stage (d0), RYBP was abundantly present in the nuclei of cells and could be also sparsely seen in the cytoplasm (figure 2*a*)*.* PLAGL1 expression was not detected at d0 and d2 time points (figure 2*a*). PLAGL1 signals were first observed from d7 and PLAGL1 gradually increased as the differentiation proceeded with highest observed expression at d14 (figure 2*a*) in agreement with the qRT-PCR results (figure 1*c*,*d*). At d7, which represents an early cardiac stage, mixed population of the cells containing both the PLAGL1-expressing and non-expressing cells were seen suggesting a non-ubiquitous expression of PLAGL1 at this stage and that the cells were probably in a heterogeneous state of differentiation (figure 2*a*).

To identify the first time point of *Plagl1* expression during cardiac differentiation, we performed gene expression analysis from samples derived between d2 and d7 time points. We performed *in vitro* cardiac differentiation (electronic supplementary material, figure S1*a*) using both wild-type and *Rybp^-/-^* mouse ES cells and collected samples every day from day 3 until day 6 (referred to as d3, d4, d5 and d6). These time points correspond to the early phase of cardiac progenitor formation when the cells undergo cardiac specification. The samples were derived for gene expression analysis of *Rybp* and *Plagl1* using qRT-PCR and protein analysis by ICC and Western blot. From our results, qRT-PCR analysis showed that in the wild-type cultures, *Rybp* expressed persistently between d0 and d6 whereas *Plagl1* expression elevated for over threefolds at d4, and the expression levels increased gradually at d5 and d6 (figure 2*b*,*c*). As expected *Plagl1* expression was not observed at any time point in the *Rybp^-/-^* cultures. Western blot analysis revealed detectable PLAGL1 from d3 correlating to its mRNA levels (electronic supplementary material, figure S1*d*). The expression kinetics of *Hymai* and *Plagl1it* mRNA was similar to that of *Plagl1* with an increase in expression levels from d3 and over threefold increase in their expression levels at d6 in the wild-type cells. The expression of *Hymai* and *Plagl1it* mRNAs was less in the *Rybp^-/-^* cultures in comparison to the wild-type (figure 2*d*,*e*). We next checked the expression kinetics of cardiac progenitor markers *Nkx2-5* and *Mef2c* to determine possible expression changes in the *Rybp^-/-^* and wild-type cells. As expected in the wild-type cultures, the expression kinetics of *Nkx2-5* increased gradually after d3 and resembled to the expression kinetics of *Plagl1* whereas *Mef2c* displayed about five-time fold increase only by d6 (figure 2*f*,*g*). In the *Rybp^-/-^* cultures, there were only subtle differences in the expression levels of *Nkx2-5* (d0-5 more and d6 less in the mutant) and *Mef2c* expressed at reduced levels at d6 in comparison to the wild-type cells.

ICC analysis was performed to see if RYBP and PLAGL1 were co-localized in the wild-type cells between d0 and d6 of cardiac differentiation, in the time window when PLAGL1 expressed first in differentiating CMCs. Our results revealed that RYBP was detected abundantly in the outgrowth of the attaching EBs after d3 (figure 2*h*). PLAGL1 was more explicitly detected from d4 in the wild-type cells, which corresponds to the early progenitor formation stage of differentiation (figure 2*h*; electronic supplementary material, figure S1*d*). The expression of PLAGL1 gradually increased from day 4 and more PLAGL1 positive cells were detected in d5 and d6 (figure 2*h*; electronic supplementary material, figure S1*d*). At d4, RYBP and PLAGL1 were co-localized in the nuclei of cells and the intensity of the PLAGL1 signal varied suggesting a heterogeneous population of cells during differentiation. The PLAGL1-expressing cells were found in the outgrowth of the attaching EBs from where differentiation is expected to proceed (figure 2*h*).

These data suggested that *Plagl1* expression was first induced during the cardiac progenitor formation stages and both RYBP and PLAGL1 prominently co-expressed in the differentiating CMCs (figure 2*a*,*c*,*h*; electronic supplementary material, figure S1*d*).

### 2.3. RYBP activates the Plagl1 P1 and P3 promoters in a polycomb-independent manner

**Figure 3.**
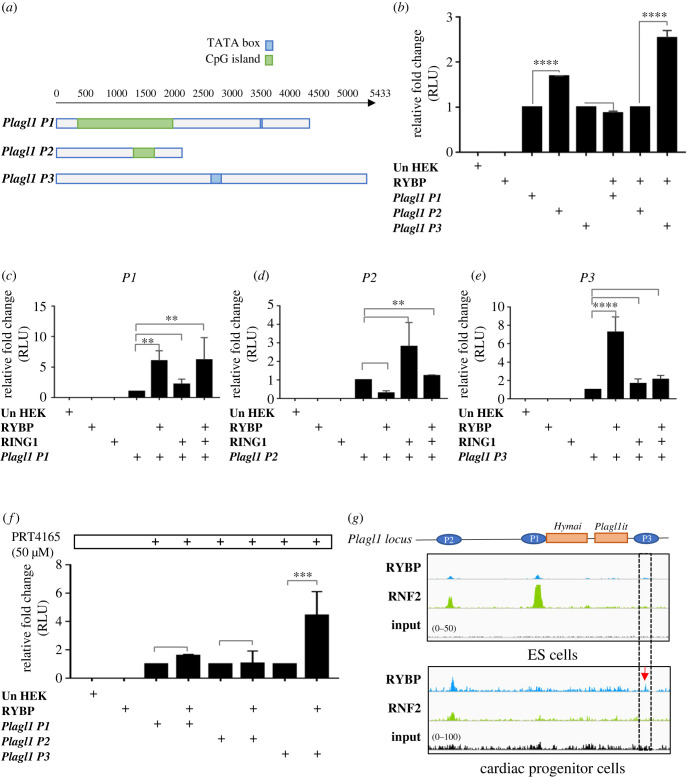
RYBP activates *Plagl1 P1* and *P3* promoters in a polycomb-independent manner. (*a*) *In silico* analysis of the *Plagl1 P1*, *P2* and *P3* promoters showing the presence of CpG islands (green) and TATA box (blue). (*b*) Luciferase reporter assay from HEK293 cells co-transfected with RYBP and luciferase-expressing constructs containing *Plagl1 P1*, *P2* or *P3* promoters. (*c*–*e*) Luciferase reporter assay from HEK293 cells co-transfected with RYBP, RING1 and luciferase-expressing constructs containing *Plagl1 P1* (*c*), *P2* (*d*) or *P3* (*e*) promoters. (*f*) Luciferase reporter assay of 50 µM of PRT4165-treated HEK293 cells co-transfected with RYBP and luciferase-expressing constructs containing *Plagl1 P1*, *P2* or *P3* promoters. Values are expressed as fold changes of luciferase activity normalized to *P1*, *P2* or *P3* single-transfected signals. (*g*) Peak derived from ChIP-seq data from GSM4052119 for RYBP (blue) and GSM4052135 (green) for RNF2 in ES cells and GSM1657391 for RYBP (blue) and GSM1657390 for RNF2 (green) in cardiac progenitor cells. The data range of the peaks is adjusted to the input GSM4052103 in ES cells (0–50) and GSM1657391 in cardiac progenitor cells (0–100) as indicated.

In order to get insights about the potential regulatory activities of RYBP at the *Plagl1* genomic locus, we next characterized the promoter regions of the *Plagl1* genomic locus in detail. Bioinformatic analysis for regulatory elements in the *Plagl1* promoters revealed that the *P1* promoter has a 1673 bp long CpG island and a 16 bp long TATA box (figure 3*a*). The *P2* promoter has a 321 bp long CpG island and has no TATA box associated with the promoter. The *P3* promoter has 70 bp long TATA box and has no CpG islands (figure 3*a*). These analyses revealed the distinct nature of the three promoters containing different regulatory elements in the *Plagl1* locus.

To examine if RYBP can activate the *Plagl1* promoters, we used a 4.6 kb *P1* promoter (−831 to 3769 from TSS of exon 4), 1.8 kb *P2* promoter (−903 to 906 from TSS of exon 1) and 5.4 kb *P3* promoter (−5211 to 161 from TSS of exon 10) from the *Plagl1* locus. Luciferase reporter assays using constructs containing *Plagl1 P1*, *P2* and *P3* promoters in *pGL4.20* vectors and the RYBP cDNA construct [22] were co-transfected in HEK293 cells (see Material and methods). Our results showed that the luciferase activity of the *P1* and *P3* promoters increased for up to 1.5 and 2.5 folds in the presence of RYBP whereas no significant change was seen with the *P2* promoter in comparison to the activity of the promoters without RYBP transfection (figure 3*b*). To determine if RYBP can activate the *P1* and *P3* promoters in a polycomb-dependent manner, we checked the inducibility of the three promoters by co-transfecting them in combination with RYBP and its core polycomb partner RING1. Our results revealed that RING1 and RYBP could not synergistically increase the expression of *P1* and *P3* promoters (figure 3*c*,*e*). In the *P2* promoter, RING1 could itself activate the promoter and small increase was also seen in the promoter activity in combination with RYBP (figure 3*d*). To further underpin the polycomb-independent activity of RYBP in activating the *P1* and *P3* promoters, we performed luciferase reporter assays by co-transfecting HEK293 cells with *P1*, *P2* and *P3* promoters together with *Rybp* cDNA construct and PRC1 inhibitor (PRT4165) [23,24]. PRT4165 inhibits the E3 ubiquitin ligase activity of the RING1/RNF2 proteins, thereby inhibiting the H2ak119ub1 activity of RING1/RNF2-containing polycomb complexes [23]. Our results showed that by co-transfecting with RYBP both *P1* and *P3* promoters maintained increased luciferase levels after treatment with PRT4165 compared to the activity of single-transfected promoters suggesting a polycomb-independent activation of the *P1* and *P3* promoters. The *P1* promoter showed 1.5-time fold increase and the *P3* promoter displayed a fourfold increase in comparison to the base P1 promoter luciferase levels whereas the *P2* promoter activity remained unchanged (figure 3*f*).

Results above suggested that *Plagl1* is a binding target of RYBP. Therefore, we compared our results to the available Chromatin immunoprecipitation (ChIP) data to see whether the *Plagl1* promoter can be possibly regulated by RYBP. ChIP-seq data were used from NCBI Geo database for the binding targets of RYBP and its polycomb cofactor RNF2 in mouse ES cells GSE42466 [4], GSE76823 [25] and in mouse cardiac progenitor cells GSE67868 [6]. The analysis revealed that both RYBP and RNF2 were bound at the *P1* and *P2* promoters in ES cells (figure 3*g*). No binding peaks for RYBP and RNF2 were observed at the *P3* promoter (figure 3*g*) in the ES cells. In the cardiac progenitor cells derived from *in vitro* differentiation of mouse ES cells, RYBP and RNF2 remained bound at the *P2* promoter (figure 3*g*). At the *P1* promoter, both RYBP and RNF2 displayed weak binding. RYBP was bound at the *P3* promoter (figure 3*g*; indicated in red arrow) in the cardiac progenitor cells and no RNF2 binding was seen at this promoter indicating a polycomb-independent regulation of the *P3* promoter by RYBP. This observation is in agreement with the obtained results from luciferase reporter assays using RING1 and the PRC1 inhibitor PRT4165 (figure 3*b–f*).

These results established that RYBP activates *Plagl1* expression via its *P1* and *P3* promoters in a polycomb-independent manner and encouraged us to assess further possible mechanisms of the activation by RYBP on the *Plagl1* locus.

### 2.4. Hymai and Plagl1it ncRNAs affect Plagl1 promoter regulation but do not synergistically function with RYBP for activation of P1 and P3 Plagl1 promoters

To determine the mechanism by which RYBP activates the *Plagl1 P1* and *P3* promoters, we investigated if RYBP activated the *Plagl1* promoters via E2Fs and YY1 binding sites. We performed luciferase reporter assays by co-transfecting *P1*, *P2* and *P3* promoter constructs in combination with RYBP, E2F2, E2F3 and YY1 overexpression constructs. Our results showed that E2F2, E2F3 and YY1 could not elevate the activation levels of *P1* and *P3* promoters by RYBP (electronic supplementary material, figure S2*a* and S2*c*). In the case of the *P1* promoter, E2F2 could induce high level of activation. *P1* promoter activity did not exhibit any statistical differences when cells were transfected with only E2F3, YY1 and in different combinations of RYBP, E2F2, E2F3 and YY1 overexpression vectors (electronic supplementary material, figure S2*a*). As expected, *P2* promoter activity decreased when cells were co-transfected with RYBP. Intriguingly single transfections and combinations of RYBP, E2F2, E2F3 and YY1 overexpression all resulted in the activation of the *P2* promoter (electronic supplementary material, figure S2*b*). In the *P3* promoter, single transfection with RYBP resulted in the highest activation of *P3* promoter, and this activation was not increased with the presence of E2F2, E2F3 or YY1 (electronic supplementary material, figure S2*c*).

**Figure 4.**
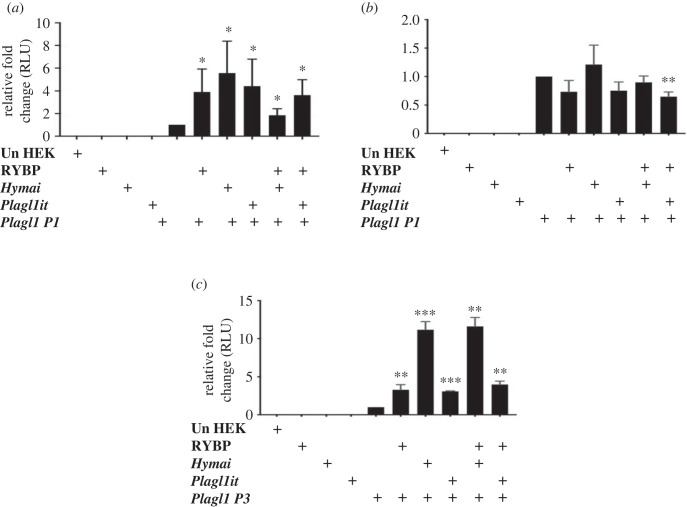
*Hymai* and *Plagl1it* did not enhance the expression levels of *P1* and *P3* promoters by RYBP. Luciferase reporter assay demonstrated that *Hymai* and *Plagl1it* cannot elevate the expression level the of the *Plagl1* (*a*) *P1*, (*b*) *P2* and (*c*) *P3* promoters when co-transfected with RYBP. Values are expressed as fold changes of luciferase activity normalized to *P1*, *P2* or *P3* single-transfected signals for (*a*), (*b*) and (*c*), respectively. The presented values are averages of three independent experiments; error bars indicate s.d. Values indicated by asterisks significantly differed from the value taken as 1 according to the statistical method one-way ANOVA (**p < 0.01; ^#^*p* < 0.0001).

We further dissected the possible mechanism by which RYBP activates *Plagl1 P1* and *P3* promoters, considering the potential contribution of the ncRNAs located in the *Plagl1* genomic locus. Since the ncRNAs in the *Plagl1* locus *Hymai* and *Plagl1it* showed similar expression kinetics to *Plagl1* during *in vitro* cardiac differentiation, we hypothesized that the two ncRNAs can synergistically function with RYBP. To test this, we amplified *Hymai* and *Plagl1it* in PCR reaction from d14 differentiated wild-type cDNA and both of them were cloned into pcDNA3.1 overexpression vector (see Material and methods) (electronic supplementary material, figure S3*a* and S3*b*). HEK293 cells were transiently transfected with the *P1*, *P2* and *P3* luciferase constructs in combination with RYBP, *Hymai* and *Plagl1it* overexpression. Luciferase assays was performed as described earlier (see Material and methods). Our results showed that neither *Hymai* nor *Plagl1it* could synergistically act with RYBP to enhance the activation levels on the *Plagl1 P1* and *P3* promoters (figure 4*a*–*c*) *Hymai* (*P1*: 5.5-fold and *P3*: 11-fold) and *Plagl1it* (*P1*: 4.42-fold and *P3*: 3-fold) exert activation compared to the activation of RYBP alone (*P1*: 4-fold and *P3*: 3-fold) (figure 4*a*,*c*). The activity of luciferase reporters driven by the *P1* and *P3* promoters was not increased in combination with RYBP and *Hymai* or RYBP and *Plagl1it* when compared to the effects induced by just *Hymai* and *Plagl1it*. On the *P2* promoter, the two ncRNAs displayed no significant changes in combination with RYBP either (figure 4*b*). These results suggested that the two ncRNAs did not affect the *P2* promoter activity. Our results also demonstrated that *Hymai* and *Plagl1it* did not affect the regulation of the *P1* and the *P3* promoters synergistically with RYBP.

### 2.5. RYBP activates the P3 promoter via Nkx2-5 consensus sites

**Figure 5.**
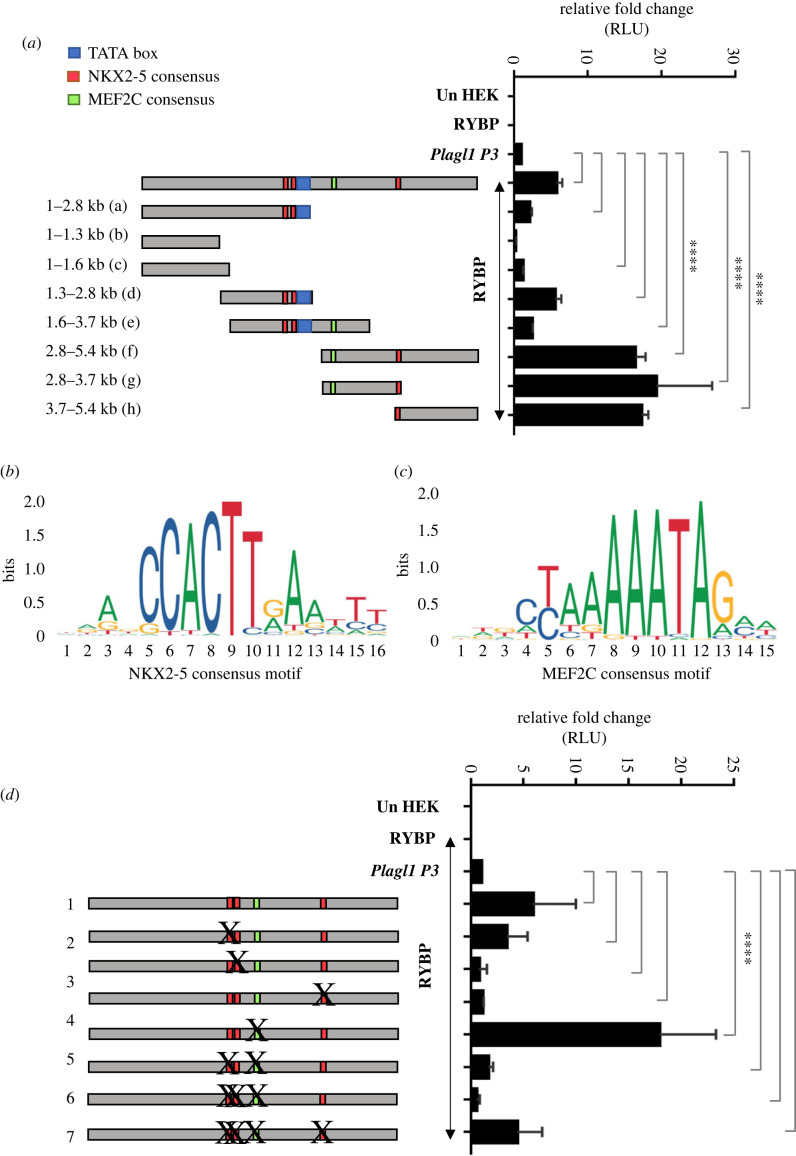
NKX2-5 consensus sites are required for the activation of the *Plagl1* P3 promoter by RYBP. (*a*) Schematic representation of the deletion mutants of *Plagl1 P3* promoter and the luciferase reporter assay of the constructs determining their inducibility by RYBP. The identified NKX2-5 and MEF2C consensus sites are indicated in green and orange bars, respectively. The position of the TATA box is indicated in blue bar. The indicative labels are on the left of the schematic representations of each mutant. (*b*,*c*) Consensus-binding sites of (*b*) NKX2-5 and (*c*) MEF2C in the 5.4 kb whole promoter region of *P3* promoter is shown. (*d*) Luciferase reporter assay using *P3* promoter mutant constructs accommodating mutation for NKX2-5 and MEF2C consensus sites and their inducibility by RYBP. Values are expressed as fold changes of luciferase activity normalized to *P3* single-transfected signals. Point mutations in the *P3* promoter are represented by X. The generated seven mutants are labelled in the left panel.

Searching further for the exact mechanism by which RYBP activates *Plagl1* expression, we have analysed possible RYBP responsive regions in the *P3* promoter. Since RYBP activated *P3* promoter the most, we made eight deletion mutants of the *P3* promoter construct and checked their inducibility by RYBP (see Material and methods). Each of the eight deletion mutants, harbouring fragments of the whole *P3* promoter, was transiently co-transfected with RYBP. Our results demonstrated that the 3′ half of the *P3* promoter (figure 5*a*–*f*,*g*,*h*) exhibited the highest activation levels by RYBP when compared to the full length and 5′ sub-clones of the *P3* promoter (figure 5*a*-a–e). RYBP does not bind to DNA directly but carries its regulatory activities via association with DNA-binding TFs such as E2F and YY1 [26].To unravel binding sites for any key cardiac TF in the 3′ region of the promoter, we performed TF binding site analysis (TRANSFAC: https://jaspar.genereg.net) and identified 3 NKX2-5 and 1 MEF2C binding sites (figure 5*b*,*c*) with the last NKX2-5 and the MEF2C site potentiating to the highest activity by RYBP (figure 5*a*).

In order to test whether RYBP acted on the *P3* promoter via the NKX2-5 and MEF2C sites, we performed luciferase reporter assays by using the mutated versions of the *P3* promoter. The three NKX2-5 and the one MEF2C binding sites were mutated to create constructs harbouring point mutations for one or more sites (see Material and methods) (figure 5*b*,*c*). HEK293 cells were transiently co-transfected with RYBP cDNA construct and the promoter constructs harbouring relevant mutations and the luciferase activity was measured (see Material and methods). Results showed that the activity of RYBP was attenuated in the mutations of the 3 NKX2-5 sites while RYBP could still activate the promoter harbouring the MEF2C mutation. These data suggested that MEF2C is not required for the activation by RYBP. The results also indicated that the NKX2-5 binding sites were required for the activation of the *P3* promoter by RYBP suggesting that RYBP might associate with NKX2-5 to activate *Plagl1* expression.

### 2.6. RYBP interacts with NKX2-5 to synergistically activate the Plagl1 P3 promoter

**Figure 6.**
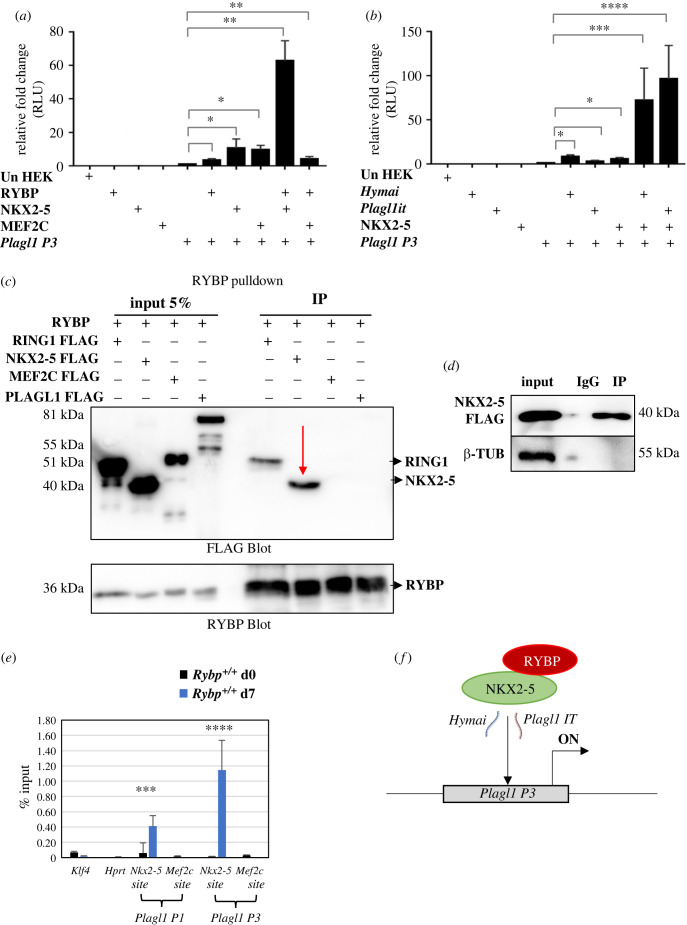
RYBP interacts with NKX2-5 and activates *Plagl1 P1* and *P3* via NKX2-5 binding sites. (*a*) The synergistic activity of RYBP and NKX2-5 at the *P3* promoter. (*b*) *Hymai* and *Plagl1it* ncRNA enhances the activation of the *P3* promoter by NKX2-5. Values are expressed as fold changes of luciferase activity normalized to *P3* single-transfected signals. (*c,d*) Co-Immunoprecipitation of RYBP with NKX2-5, MEF2C and PLAGL1 revealed that RYBP interacted with NKX2-5. RING1 was used as the positive control. (*e*) ChIP-qPCR assays by normalizing the values to input determined that RYBP bound at the *P1* and *P3* promoters at the NKX2-5 binding sites. (*f*) Schematic representation of the activation of *Plagl1 P3* promoter by RYBP and NKX2-5 in cooperation with *Hymai* and *Plagl1it*.

To understand how the NKX2-5-binding sites affected the regulation of the *P3* promoter by RYBP, we next investigated if RYBP can work together with NKX2-5. Luciferase reporter assays were performed by co-transfecting the *P3* promoter in combination with RYBP and either NKX2-5 or MEF2C overexpression constructs. Our results revealed that NKX2-5 could itself activate the *P3* promoter up to 10-fold and that the expression level was further increased up to 60-fold when RYBP was also present (figure 6*a*). MEF2C overexpression could also activate the *P3* promoter for up to 10-fold as expected [21] but the activation level dropped in the presence of RYBP indicating that MEF2C does not function synergistically with RYBP to activate the *P3* promoter (figure 6*a*). We also checked if *Hymai* and *Plagl1it* ncRNAs could enhance the activation ability of RYBP and NKX2-5 together at the *P3* promoter (figure 6*b*). Our results showed that both *Hymai* and *Plagl1it* could not only synergistically enhance the activation of the *P3* promoter by NKX2-5, but also were able to maintain high expression levels in the samples transfected with both RYBP and NKX2-5 (figure 6*b*; electronic supplementary material, figure S3c). These results indicated that both *Hymai* and *Plagl1it* synergistically functioned with RYBP and NKX2-5.

Since these results indicated a potential interaction between RYBP and NKX2-5 we next performed co-immunoprecipitation (Co-IP) experiments by co-transfecting HEK293 cells with RYBP in combination with NKX2-5, MEF2C and PLAGL1 (see Material and methods). We used RING1 as a positive control since RING1 is a known interactor of RYBP [8]. Our results showed that RYBP interacted with NKX2-5 (figure 6*c*,*d*) but did not interact with MEF2C and PLAGL1.

To verify if NKX2-5 binds at the *Plagl1* promoters during cardiac development, available ChIP-seq data from mouse ES cell-derived cardiac progenitor cells (GSM2054327) and terminally differentiated CMCs (GSM2054330) were analysed. NKX2-5 bound at both the *P1* and *P3* promoters in both stages of *in vitro* cardiac differentiation (electronic supplementary material, figure S5*a* and S5*b*).

These results indicated that RYBP might mediate its effects via NKX2-5 on the *Plagl1 P3* promoter. Therefore, we next performed ChIP assay coupled with qRT-PCR to check if RYBP could bind at the *Plagl1 P3* promoter via the NKX2-5 sites. ChIP assay was performed with sheared chromatin collected from wild-type ES cells and d7 differentiated CMCs (see Material and methods). The primers specific to NKX2-5 sites at the *P1* and *P3* promoters were designed to amplify between 80 and 120 bp encompassing the corresponding consensus sites (electronic supplementary material, table S3). The sheared chromatin was immunoprecipitated with magnetic beads coated with RYBP antibody, and the immunoprecipitates were carefully eluted. 1% of the sheared chromatin was used as input. QRT-PCR was performed using HPRT as the negative control. From our results, RYBP bound to the NKX2-5 sites of both *P1* and *P3* promoters indicating the interaction between RYBP and NKX2-5 is important for the activation of *Plagl1 P1* and *P3* promoters via the consensus-binding site for NKX2-5 (figure 6*e*,*f*).

### 2.7. PLAGL1 is a potential regulator of sarcomeric gene expression by activating *Tnnt2* promoter

**Figure 7.**
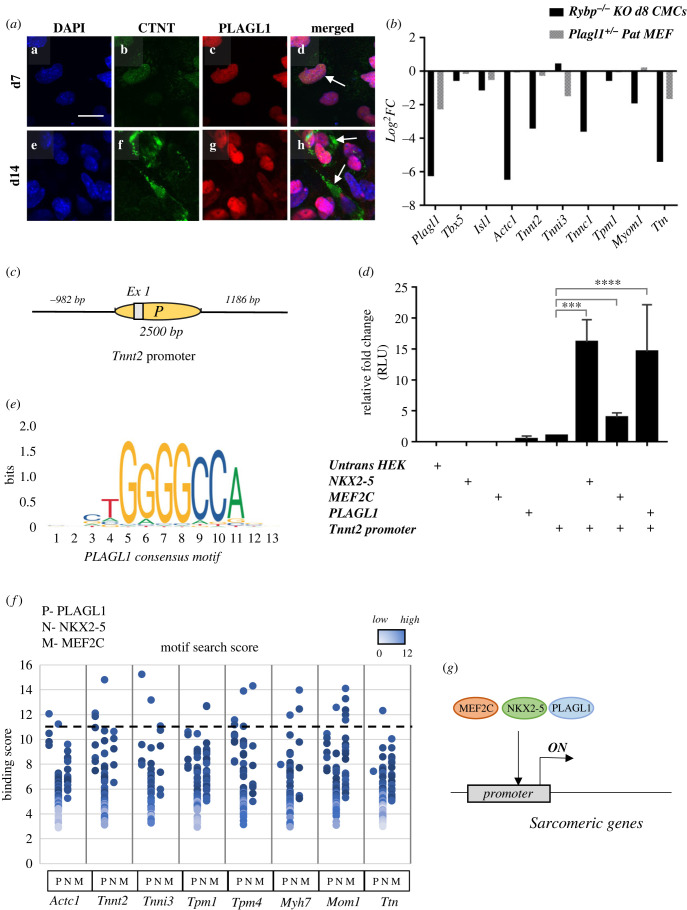
PLAGL1 co-expressed with CTNT and activates the *Tnnt2* promoter. (*a*) ICC analysis of PLAGL1 and CTNT in d7 and d14 CMC displaying the co-expression of PLAGL1 and CTNT in differentiating CMCs. White arrows indicate the cells with strong PLAGL1 and CTNT co-expression. Immunostainings: Blue: DAPI (nuclei), green: CTNT, red: PLAGL1. Olympus Confocal IX 81, obj: 180×. Scale bar: 100 µm. (*b*) Transcriptional changes in key sarcomeric genes in d8 *Rybp* null mutant cardiac differentiated cells and *Plagl1* KO MEF cells. (*c*) Schematic representation of the position of the *Tnnt2* promoter. (*d*) Luciferase reporter assay to determine the inducibility of *Tnnt2* promoter by PLAGL1. NKX2-5 and MEF2C were used as the positive controls. (*e*) Consensus-binding motif of PLAGL1 according to JAPSAR. (*f*) Motif search for PLAGL1, NKX2-5 and MEF2C in sarcomere genes *Actc1*, *Tnnt2*, *Tnni3*, *Tpm4*, *Myh7*, *Myom1* and *Ttn*.

In order to get insights into the biological functions of PLAGL1, we asked if the expression of PLAGL1 specific to the formation of a particular cell type during *in vitro* cardiac differentiation. ICC analysis by co-staining d7 and d14 wild-type CMCs was performed by using anti-PLAGL1 antibody and markers for cardiac endothelial (GATA4), epithelial to mesenchymal transition and microtubule intermediate filament (VIMENTIN), neurofilament (2H3), smooth muscle (SMMHC) and CMC (cardiac troponin T2 (CTNT)) (electronic supplementary material, figure S6*a*). Our results showed that PLAGL1 was co-stained with CTNT (figure 7*a*), underlining its role in CMC development. PLAGL1 was not present in endothelial lineages (electronic supplementary material, figure S6*a*). PLAGL1 was present in some cells expressing VIMENTIN and SMMHC and in neural lineages (electronic supplementary material, figure S6*b*–*d*). These results indicated a possible role of PLAGL1 in the formation of several lineages including neurofilaments and CMCs.

In order to examine if the lack of PLAGL1 in the *Rybp^-/-^* CMCs affected the formation of terminally differentiated CMCs, we compared the gene expression of sarcomere genes in the *Plagl1* null mutant (*Plagl1* KO) mouse embryonic fibroblasts (MEF) cells (GSM2643646) and our previously reported whole-genome transcriptome from d8 differentiated cardiac cultures from the *Rybp* null mutant. This analysis revealed that several components of the sarcomere, such as *Tnnt2* (*Rybp^-/-^*: −3.42; *Plagl1* KO: −0.26) and *Ttn* (*Rybp^-/-^*: −5.41; *Plagl1* KO: −1.66) were downregulated in both at the d8 *Rybp* null mutant CMCs and in the *Plagl1* KO MEF cells (figure 7*b*), which indicated a possible connection between the expression of *Plagl1* and regulation of sarcomere genes.

To assess whether PLAGL1 can transcriptionally regulate sarcomere genes, we amplified and cloned the 2500 bp long promoter region of *Tnnt2* (−982 to 1689 from the ATG) into luciferase reporter containing vector (electronic supplementary material, figure S7) (see Material and methods). Luciferase reporter assays were performed by co-transfecting HEK293 with *Tnnt2* promoter construct and overexpression constructs for either PLAGL1, NKX2-5 or MEF2C (see Material and methods). NKX2-5 and MEF2C were used as positive controls since their expression has been previously shown to regulate sarcomere genes [27,28]. Our analyses revealed that PLAGL1 can activate the *Tnnt2* promoter (figure 7*c*). As expected NKX2-5 activated the *Tnnt2* promoter up to 15 folds and MEF2C activated the promoter fourfolds compared to the activity of the *Tnnt2* promoter itself without NKX2-5 or MEF2C transfection. Importantly, PLAGL1 could activate the *Tnnt2* promoter for over 20-fold indicating the role of PLAGL1 in activating *Tnnt2* expression.

By performing TF binding analysis for the identified PLAGL1 consensus GGG(G/C)(G/C)CC motif and the consensus-binding sites for NKX2-5 and MEF2C (https://jaspar.genereg.net), we found that PLAGL1 has binding motif in sarcomere thin filament marker genes such as: *Actc1*, *Tnnt2*, *Tnni3*, *Tpm4* and sarcomere thick filament markers: *Myh7*, *Myom1* and *Ttn* (figure 7*f*,*g*; electronic supplementary material, figure S7). Based on these results, we suggest the possible role of PLAGL1 during *in vitro* cardiac differentiation by regulating sarcomere genes, thus in the contractility of CMCs.

## 3. Discussion

Our results demonstrated that RYBP activated *Plagl1* via its *P3* promoter and that this activation was mediated by a key cardiac TF NKX2-5. We also showed that this activation ability of RYBP is independent of its polycomb core functions. To our knowledge, this is the first demonstration, that RYBP together with NKX2-5 is able to activate a cardiac TF, which highlights the ncPRC1-independent activator functions of RYBP. Our study further suggests the possible role of ncRNAs in cardiac development and disease formation.

RYBP is a ‘moonlighting’ protein, thus able to interact with multiple proteins with diverse biological functions. RYBP, as a crucial, core component of the ncPRC1s, were mostly highlighted for its role as a repressor [8]; however, in the past years, ncPRC1.3 and ncPRC1.5 were also identified to activate genes related to autism in the CNS [29]. In these ncPRCs several newly identified partners, e.g. AUTS2, p300 and CK2 could convert the repressive function to transcriptional activation. Furthermore, impairment in AUTS2 and P300 interaction was found in developmental disorders, including Rubinstein-Taybi syndrome [30,31]. The mutation of AUTS2 resulted in misregulation of target developmental genes hampering normal motoneuron formation. These observations emphasized further a critical role of ncPRC1 subunits and their interactors in differentiation and disease development. Also, these results rose the question whether RYBP had been acting as core member of the ncPRC1s to activate *Plagl1* or alternatively, acting independently from ncPRC1s. One example of the latter when RYBP could act a bridging factor between TFs E2F2 or E2F3 and YY1 in order to activate *Cdc6*, a gene required for early steps of DNA replication [26]. However, in our experiments, RYBP did not act in synergy with any E2Fs to activate the *Plagl1* promoter (electronic supplementary material, figure S2*a*–*c*), which suggested that RYBP regulated *Plagl1* not only in a ncPRC1 but also in an E2F independent manner.

We have also determined that only the *P1* and *P3* promoters were active during the time course of *in vitro* cardiac differentiation (figure 1*a*–*d*). Several previous works were focused at characterizing the *Plagl1* promoters in the context of imprinting. In these studies, unusual biallelic expression of *Plagl1* from an alternate promoter *P2* situated 30 kb upstream to the *P1* promoter (site of imprinting) was described in patients with TNDM [20]. Later studies have also identified the presence of a novel alternate *P3* promoter which lies immediately upstream to the start codon in exon 10 of the *Plagl1* locus [21]. Our results revealed that RYBP activates both the *P1* and *P3* promoters and that this function of RYBP is polycomb-independent (figure 3*b*–*f*). In silico methylation analyses of the *Plagl1* locus in ES cells and cardiac progenitor cells revealed repressive histone methylation marks (H3K27me3 and H3K4me3) at the *P1* and *P2* promoters in ES cells and CMCs and weak activation marks at the *P3* promoter in CPCs (electronic supplementary material, figure S5*c* and S5*d*). These data further strengthen that the *Plagl1 P2* promoter is active in specific tissues such as leucocytes and pancreas during disease states such as TNDM [20,32,33] and not during cardiac development.

By combining transcription factor binding site (TFBS) analyses and using truncation mutants of the *P3* promoter, we also determined that the consensus-binding site for cardiac TF NKX2-5 was required for the activation of the *P3* promoter by RYBP (figure 5*a*). NKX2-5 was previously determined to activate the expression of *Plagl1* in mouse hearts [16]. It was also shown that in human patients with mutations in *Nkx2-5* often have arrhythmias [34,35]. ChIP-seq analysis of NKX2-5 binding in cardiac progenitor cells and CMCs revealed that NKX2-5 bound at the *P1* and *P3* promoter in both cardiac progenitor cells and CMCs (electronic supplementary material, figure S5*a* and S5*b*). By performing site-directed mutagenesis of the NKX2-5 consensus sites, we confirmed that the NKX2-5 consensus sites were essential for the activation of the *P3* promoter by RYBP (figure 5*d*). Other cardiac TFs such as GATA4, MEF2C and serum response factor also bound at the *P1* and *P3* promoters (electronic supplementary material, figure S5*a*) further indicating the activity of *P1* and *P3* promoters during cardiac differentiation in accordance with our gene expression analysis (figure 1*b*–*d*).

We also demonstrated that RYBP interacts with NKX2-5 at the protein level (figure 6*c*), and this interaction is important in the regulation of *Plagl1* in the wild-type CMCs. RYBP was bound at the NKX2-5 consensus sites in both *P1* and *P3* promoters at d7 CMCs when *Plagl1* is normally expressed and not at d0 pluripotent stage (figure 6*e*). NKX2-5 is necessary for progenitor formation and expressed from the cardiac lineage commitment stages (electronic supplementary material, figure S4), in correlation to the expression kinetics of *Plagl1* during cardiac differentiation. Interestingly, PLAGL1 itself can interact with NKX2-5 in order to regulate the ANF promoter [16] suggesting a complex interplay among cardiac TFs during heart development.

Previous studies have established the expression of *Plagl1* in mouse cardiac crescent from E7.5 and stronger expression in the heart myocardium from E8.5 [16]. Our study revealed the expression kinetics of *Plagl1* during the time course of *in vitro* cardiac differentiation of ES cells. This observation has a particular relevance as we have no information about what the exact function of *Plagl1* is during mammalian cardiac development. Publications established that *Plagl1* was expressed in a chamber-restricted pattern in the mouse embryonic heart and was often mutated in CHDs [16,36]. In our experimental system, the expression of *Plagl1* was first detected at day 4, which is an early stage when the cardiac progenitors form indicating a potential novel role *of Plagl1* in early lineage commitments (figure 2*c*; electronic supplementary material, figure S1*d* and S1*e*). Proliferation and differentiation of CMCs is disturbed in several left ventricle hypoplasia or hypoplastic left heart syndrome and contributing to the development of CHD [37]. As of tissue specificity, *Plagl1* was expressed in CMCs but not in the endothelial cells during *in vitro* cardiac differentiation of mouse ES cells. *Plagl1* was shown to be expressed in mouse placentas and knockdown on *Plagl1* by siRNA in human placentas resulted in decreased expression of genes associated with placental vasculature development [38]. One explanation of the different expression of *Plagl1* in endothelial lineages could be that *Plagl1* may have different tissue specificity and different functions in the placentas than in the embryo. It is also worth to be noted that although in our experiments *Plagl1* expression was not present in endothelial cells but it was often adjacent to them. This suggests that *Plagl1* may have role in signalling to neighbouring cells via the formation of epithelial-mesenchymal transitions (EMT). This was indeed confirmed by functional enrichment analyses when *Plagl1* expression was associated with EMTs in human cervical cancer samples (electronic supplementary material, figure S6*a*).

Our results also indicated a potential interaction of *Hymai* and *Plagl1 IT* with NKX2-5 as the ncRNAs increased the fold activation levels of the *P3* promoter by NKX2-5 extensively (electronic supplementary material, figure S3*c*) suggesting an extended network of regulators involved in cardiac development. Several ncRNAs have been identified to play vital roles in cellular processes, including chromatin remodelling, DNA repair and translation [39]. NcRNAs inactive X-specific transcripts (*Xist*), braveheart long ncRNA (*Bvht*) and maternally expressed gene 3 (*Meg3*) have also been identified to interact with or inhibit PRC members [40–43]. *Bvht* and *Meg3* were also shown to induce cardiac lineage commitment and are expressed upstream to *Mesp1* with the potentiate to regulate a core cardiac gene network. [41,43]. NcRNAs control functions of various cells of the heart including migration, proliferation, angiogenesis and their misregulation occur in many tumours as well as in non-oncogenic diseases. Due to their versatile roles during heart formation, ncRNAs are subjects for developing new diagnostic and therapeutic tools as well [44]. However, their role in the development of CHD is not well understood. The biological functions of the two ncRNAs at the *Plagl1* genomic locus *(Hymai* and *Plagl1it)* are not known, a few gene expression studies mention their overexpression related to diseases. Both *Hymai* and *Plagl1it* are imprinted and expressed only from one allele from the *Plagl1* genomic locus [45]. The expression of *Hymai* is partially connected to the expression of *Plagl1* since *Hymai* is also transcribed upon the regulation of *P1* promoter. Altered expression of both *Plagl1* and *Hymai* were described as indicative of disease condition such as TNDM and tumours [46,47]. In our experiments, altered expression pattern of both *Hymai* and *Plagl1it* was recorded in the *Rybp* null mutant cardiac cultures (figure 1*g*,*h*) suggesting that the lack of RYBP influences their expression and this might be related to disease conditions as well*.* High expression level of the two ncRNAs at d14 of cardiac differentiation in the wild-type CMCs suggested that the ncRNAs might function during the terminal stages of CMC formation (figure 1*g*,*h*). We have also revealed that the two ncRNAs were able to activate the *Plagl1* promoters. However, the overexpression of either *Hymai* or *Plagl1it* did not alter the activity of the *Plagl1* promoters when RYBP was also transfected (figure 4*a*–*c*) suggesting that RYBP works independently of the ncRNAs. Another intriguing question is whether the two ncRNAs can potentially enhance the transcriptional initiation ability of NKX2-5 or they initiate the transcription of *Plagl1* independently from NKX2-5. Further studies will need to clarify the promoter/enhancer region corresponding to *Plagl1it* and study the exact mechanism how *Plagl1it* can regulate cardiac development at normal and disease conditions.

Our study further suggested that the lack of *Plagl1* in the *Rybp^-/-^* CMCs can be one possible causative of the uncontractile phenotype. The *Rybp* null mutant CMCs do not have proper sarcomere and subsequent contractility [14]. The formation of proper sarcomeres is indispensable for the contractility of CMCs. Impaired sarcomere activity is implicated to various heart disorders including arrhythmia. The regulation of sarcomere components is directly connected to the expression of key cardiac TFs [48], however, much remains unclear about the mechanisms that regulate the expression of cardiac TFs and the consequent effects on sarcomere activity. PLAGL1 is expressed abundantly in mouse embryonic myocardium and *Plagl1* disruption caused atrial, ventricular septal defects, thin ventricular walls and impaired heart functions [16]. These suggested PLAGL1 can function in the regulation of sarcomere. PLAGL1 staining was profoundly present in the CTNT-positive cells, suggesting that PLAGL1 expressed in cells differentiating towards terminal CMCs (figure 7*a*). Indeed, our luciferase reporter assays demonstrated that *Plagl1* was able to activate the expression of the mouse *Tnnt2* promoter. The activation of the *Tnnt2* promoter is essential for CMC development and contractility. Since *Plagl1* expression was not detected neither at mRNA nor at protein levels in the *Rybp* null mutant cultures, the loss of *Plagl1* functions could, at least partially contribute to the phenotype of the *Rybp* null mutant CMCs. This may be manifested via the lack of *Tnnt2* activation, which need to be addressed in further studies.

As of possible role of PLAGL1 in lineage commitment we could establish that PLAGL1 is unlikely to be required for cardiac endothelial formation but could function in the formation of mesenchymal derived cell types including smooth muscle cells and CMCs (figure 7*a*; electronic supplementary material, figure S6). PLAGL1 co-staining with cells for neurofilament-specific markers indicated that PLAGL1 might have a possible role in the cardiac conduction system (electronic supplementary material, figure S6C) [49,50]. During the cardiac progenitor formation, the expression of T-Box proteins T-Box 3 (*Tbx3*), T-Box 5 (*Tbx5*) and T-Box 18 (*Tbx18*) are required for the generation of pacemaker cells that function in the conduction system of the heart [51]. As a result of these finely tuned events governed by series of key TFs, the developing heart starts beating as early as E7.5–8 in mouse [52]. Further studies need to address whether PLAGL1 can regulate genes required for pacemaker cell development or PLAGL1 is important for lineage commitment steps during cardiac progenitor formation. In fact, important functions of PLAGL1 in neural development have been established [53,54]. In the *Plagl1*, null mouse neocortical progenitors proliferate less and instead produce more neurons and misexpression of *Plagl1* interferes with normal neural differentiation. *Plagl1* misexpression also blocked neuronal migration, with *Plagl1*-overexpressing neurons pausing more frequently and forming fewer neurite branches during the period when locomoting neurons undergo dynamic morphological transitions. Similar, albeit less striking, neuronal migration and morphological defects were observed on *Plagl1* knockdown, indicating that *Plagl1* levels must be regulated precisely.

**Figure 8.**
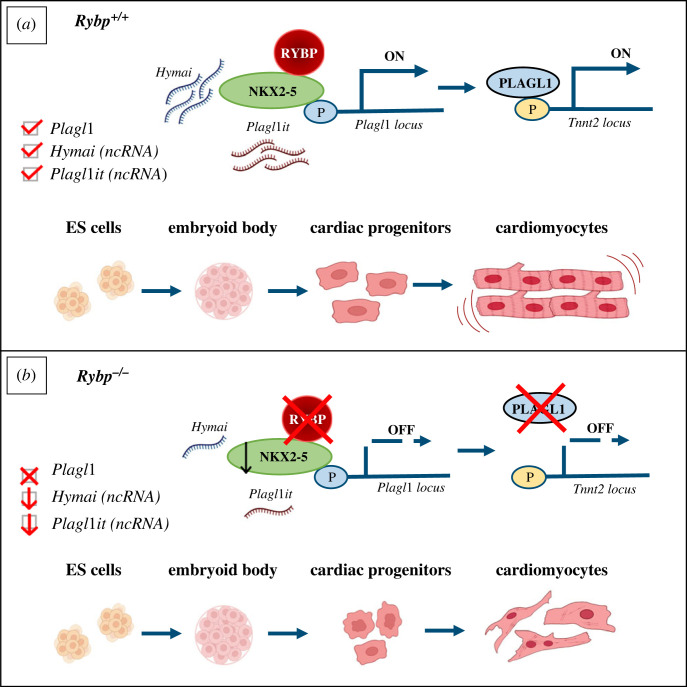
RYBP activates *Plagl1* in the wild-type but not in the mutant cells. *(a)* When RYBP is present, RYBP interacts with NKX2-5 to activate *Plagl1* expression. Abundant expression of *Plagl1* together with NKX2-5, *Hymai* and *Plagl1it* ncRNAs promote CMC formation and contractility. (*b*) In the absence of RYBP, *Plagl1* is not expressed affecting sarcomere formation and contractility.

Our results provide a novel understanding about the role of *Plagl1* in CMC formation and the molecular mechanism by which RYBP functions during cardiac morphogenesis via starting-up *Plagl1* expression and can also give a reasonable explanation of why the *Rybp^-/-^* CMCs are not able to contract (figure 8). In the absence of *Rybp, Plagl1* is not expressed and *Hymai* and *Plagl1it expression is also* compromised resulting impaired activation of *Tnnt2* or other thin and thick filaments of the sarcomere. This can result the malfunction of sarcomeres and lead to impaired contraction of the *Rybp^-/-^* CMCs. In wild-type cells, when *Rybp* is present, there is enough amount of *Plagl1* and ncRNAs in the cells and the contractility of sarcomere filaments is not compromised, cells can form beating CMCs (figure 8).

Taken together, the interaction between RYBP and NKX2-5 proteins broadens our understanding about the alliance between PcGs and lineage-specific TFs to govern differentiation. Overall, these results also affirm the theory that in certain cases PcGs, such as RYBP could exert their roles as transcriptional activators during development.

## 4. Material and methods

### 4.1. Cell lines and culture condition

Mouse R1 ES cells [55] (mentioned as wild-type or *Rybp^+/+^*) and R1 derived *Rybp* null mutant ES cells (mentioned as *Rybp^-/-^*) [11] were thawed and on mitomycin C (Mit C; Sigma, cat. no. M0503) inactivated MEF layer and cultured as previously described by Henry *et al*., 2020.

HEK293 cells were maintained in DMEM (DMEM with 4.5 g I^−1^ glucose & L-glutamine, Lonza cat. no. BE12-604F) contained 10% FBS (Gibco, cat. no. 10500), 0.1 mM non-essential amino acids (MEM non-essential amino acids (100x), Gibco, cat. no. 11140-035), 1% sodium pyruvate (100 mM, Gibco, cat. no. 11360-039) and 50 U ml^−1^ penicillin/streptomycin (100x, Gibco, cat. no. 15140-122). The cells were passaged before the confluency reached 90% (approximately every 2–3 days). Medium was changed every second day. Cells were cultured in humidified conditions containing 5% CO_2_ at 37°C.

### 4.2. In vitro cardiac differentiation

Mouse ES cells were harvested as single suspension using 0.05% (wt vol^−1^) trypsin (Trypsin-EDTA (1x) 0,05% / 0,02% in D-PBS, Gibco, cat. no. 15400-054) and then the cell number was calculated using a burker chamber. The cell number was diluted to 50 cells µl^−1^ in suspension 20 µl droplets of cell suspension were dispensed to lids of bacterial dishes where each droplet contains around 800–1000 cells, and then the cells were let to form EBs by the HD method as described in Keller *et al*. [56]. The EBs were harvested on the second day and plated into cell culture dishes (60 mm, Corning, cat. no. 430196) coated with gelatine-containing ES medium (described in §4.1) without LIF. The medium was changed every second day and the cells were cultured up to 21 days. The cells were harvested for further analysis at different time points of cardiac differentiation: day 0, 2, 7, 10, 14 and 21 (labelled as d0, d2, d7, d10, d14 and d21). Day 0 represents pluripotent stem cell stage, day 2 represents the EB stage, day 7 and day 10 represent early and late cardiac progenitor stages respectively and day 14 and day 21 represent the terminal stage of *in vitro* cardiac differentiation.

### 4.3. Calcium phosphate transient transfection method

Calcium phosphate (CaPO_4_) method [57] was used to transiently transfect HEK293 cells for reporter assays and protein overexpression for protein stability assays and co-immunoprecipitation analysis. HEK293 cells were seeded at a density of 1 × 10^6^ cells per 6 cm tissue culture dishes and maintained as described above. Five hours before transfection the cells were fed with fresh medium. The transfection mix was prepared by diluting the required plasmids in 0.1 mM Tris-EDTA (Trizma base, Sigma, cat. no. T1503) buffer and 2.5 M Calcium chloride (CaCl_2_, Sigma, C-3881) and 2X HEPES buffered saline (HBS, Sigma, cat. no. H3375) dropwise by bubbling the solution using pasteur pipette to provide oxygen for the mixture. The transfection mix was added to the cells dropwise and the cells were then maintained with the transfection mix in humidified conditions. 16 h after the transfection fresh media was provided to the cells and after 40 h the cells were washed twice with 2 ml of 1X PBS on ice and then harvested for their whole cell protein lysate using cell lysis buffer (Cell culture lysis 5X reagent, Promega, cat. no. E153).

### 4.4. The luciferase reporter assay system

HEK293 cells were transfected with CaPO_4_ transient transfection method as mentioned above. The transfected cells were harvested for their protein lysates 40 h after transfection with 1X PLB (Passive lysis buffer provided by the luciferase assay kit; Dual Luciferase Reporter Assay System, Promega, cat. no. E1910). Concentration of the whole cell lysate was determined by the Bradford's method (5X Bio-Rad Protein Assay Dye reagent concentrate, cat. no. 5000006) according to the manufacturer's instructions. Protein concentrations were measured from OD_600_ taken in UV spectrophotometer (WPA Photometer UV110 Cambridge, UK, cat. no. RS232). The concentration of the lysates was then determined by Bradford's method [58] using Bovine Serum Albumin (BSA, VWR, cat. no. G22361V) as the standard. 20 µg of the protein lysates were measured from each transfection with 100 µl of Luciferase Assay Reagent II (LAR II, provided with the kit). Luciferase activity was recorded with Pelkin Elmer TopCount NXT Luminometer in dark conditions. Each measurement was recorded in triplicates.

### 4.5. Inhibition of PRC1 activity

Inhibition of PRC1 activity was performed to analyse the PRC-dependent and independent activities of RYBP in promoter assays. Sixteen hours after transfection of the required plasmids by CaPO_4_ method (detailed in §4.3), HEK293 cells were fed with growth media supplemented with 50 µM of PRC1 inhibitor, PRT4165 (PRT4165, Sigma, cat. no. NSC600157) as previously reported by Ismail *et al*. and Gracheva *et al*. [23,24]. The cells were maintained with PRT4165 supplemented media for further 24 h after transfection and the cells were harvested for whole cell lysates. The cell lysates were then prepared for luciferase reporter assay as described in §4.4.

### 4.6. Quantitative real-time PCR

Relative quantification of mRNA expression during *in vitro* cardiac differentiation was performed using qRT-PCR. Total RNAs were isolated from the harvested cells using GeneJET RNA Purification Kit (Thermo Scientific, cat. no. K0732) according to the manufacturer's instruction. Reverse transcription PCR for the cDNA synthesis from the isolated RNA was performed using Applied Biosystems High-capacity cDNA Reverse Transcription Kit (Invitrogen Life Technologies, cat. no. 4368814 Carlsbad, CA, USA) according to the manufacturer's instructions. qRT-PCR analysis was performed in SYBR green master mix (SYBR Select Master Mix for CFX, Applied Biosystems, cat. no. 4472942) using Bioer LineGene Real-time PCR system (Bioer, China).

Relative mRNA expression changes were determined using the *ΔΔ*Ct method. The threshold cycle (Ct) values for each gene were normalized to the expression level of *Hprt* (Hypoxanthine phosphoribosyl transferase I) as an internal control. To calculate the fold expression changes the values were compared to the expression of d0 *Rybp^+/+^* ES cells. The primers used in this study are listed in the electronic supplementary material, table S2.

### 4.7. Chromatin immunoprecipitation and quantitative real-time PCR

ChIP was performed by using EpiXplore ChIP kit, Clonetech, cat. no. 632011) according to manufactures instructions. In brief, nuclear extraction from ES cells and d7 cardiac differentiated cells from 10 cm plates was carried out by carefully lysing the cytoplasm and nuclei isolation using the lysis buffers (provided in the kit) and subsequent shearing of the DNA was performed using an ultrasonicator (Ultrasonic homogenizer 3000, BioLogics) at 4 × 30 s cycles, 30 pulse and 20 kHz. The sheared DNA was loaded into 1% agarose gel electrophoresis and the size of the sheared chromatin was seen between 200 bp to 800 bp (ideal for IP and qRT-PCR). The sheared DNA was then incubated with prewashed magnetic beads (Mag Capture beads, Clonetech, cat. no. 632577) under gentle rocking for 4 h at 4°C. The wash steps were carried out according to the manufacturer's instructions with the help of a magnetic stand. The eluted immunoprecipitated chromatin was then treated with RNase A and protease.

The immunoprecipitated chromatin was then used for qRT-PCR using SYBR green as mentioned above using the primers listed in the electronic supplementary material, table S3.

### 4.8. Cloning Plagl1 P1 and P2 promoter regions, subcloning of the Plagl1 P3 promoter, cloning of Hymai, Plagl1it, Nkx2-5 and Mef2c overexpression constructs

Luciferase reporter constructs for *Plagl1 P1* and *Plagl1 P2* promoters were generated by amplifying 4600 bp region containing *Plagl1 P1* and 1809bp region of *Plagl1 P2* via PCR with the addition of HindIII restriction sites at both the 5′ and 3′. The *Plagl1 P1* promoter was amplified from −1026 from exon 4 containing a 1.6 kb CpG island including entire exon 4 and 3.4 kb from intron 4. The *Plagl1 P2* promoter was amplified from −903 from exon 1 containing a 655 bp long CpG island, entire exon 1 and 550 bp from intron 1. The promoter regions were decided based on previous publications and the position of the CpG islands. Full-length cDNA constructs of *pcDNA3.1-Hymai, pcDNA3.1-Plagl1it, pRK7-FLAG-Nkx2-5* and *pRK7-FLAG-Mef2c* were generated by amplifying their cDNA from wild-type d14 (highest expression time point) by introducing XbaI restriction sites for *Hymai* and *Plagl1it* and BamHI restriction sites for *Nkx2-5* and *Mef2c*. The orientation of the cloned cDNA constructs was confirmed by sequencing. All cloning's were performed using NEB One*Taq* Hot Start DNA Polymerase, NEB, Cat #M0481L. The primers used for cloning are listed in the electronic supplementary material, table S3.

### 4.9. Subcloning the Plagl1 P3 promoter

The subcloning of the *P3* promoter was performed as follows. Clones a (1–2.8 kb) and f (2.8–5.4 kb) were produced by cleaving the *P3* with BglII. Clone a (1–2.8 kb) was self-ligated after digestion with BglII and the 2.8–5.4 kb band was eluted and re-cloned into pGL3 empty vector at the BglII site. Clones b (1–1.3 kb) and d (1.3–2.8 kb) were generated by HindIII digestion of clone a. Clone e (1.6–3.7 kb) construct was generated by digesting the *Plagl1 P3* promoter by PstI, gel elution of the 2.1 kb band and re-cloning the fragment into the same sites in pGL3 empty vector.

Clones g (2.8–3.7 kb) and h (3.7–5.4 kb) were generated by digesting clone f with PstI and performing self-ligation and insert ligation of fragments as mentioned earlier.

### 4.10. Cloning Tnnt2 promoter

The *Tnnt2* promoter (2688 kb) was PCR amplified using wild-type gDNA from ES cells as template. The PCR amplicon was gel eluted and cloned into KpnI sites and cloned into pGL4.20 vector as described above.

### 4.11. Site-directed mutagenesis

Site-directed mutagenesis was performed using Q5 site-directed mutagenesis kit (NEB, cat. no. E0554S) following the manufacturer's instructions. Primers were designed to mutate consensus sites for *Nkx2-5* and *Mef2c* at the *P3* promoter by using NEBase Changer tool (https://nebasechanger.neb.com) provided by NEB (electronic supplementary material, table S4). The primers were designed to mutate the consensus of 3 *Nkx2-5* and one *Mef2c* sites by introducing BamHI and HindIII sites, respectively, at the consensus to assist with screening positive mutants harbouring the right mutation. The PCR reaction was set according to the corresponding primer annealing temperature suggested by NEBase Changer tool. The KLD (Kinase, ligase and DpnI digestion) enzyme (provided in the kit) was used to digest template DNA and ligation for rapid generation of mutant constructs carrying mutation for *Nkx2-5* and *Mef2c* consensus. The transformed colonies were then screened and confirmed by BamHI and HindIII digestions for *Nkx2-5* and *Mef2c* consensus sites, respectively. Seven different mutants were generated harbouring single and multiple mutants of *Nkx2-5* and *Mef2c* consensus (figure 5*d*). Further confirmation was performed by sequencing the plasmids (Deltagene, Szeged, Hungary) and checked for carrying the mutation with no off-target mutations in the constructs.

### 4.12. Luciferase reporter assay

HEK293 cells were transiently co-transfected with the following plasmids: *pGL3.Plagl1-P3-Luc* (a kind gift from Michael P. Czubryt) [21], *pcDNA3.1-HA-Rybp*. 5 µg *pGL4.Plagl1-Luc* and increasing concentrations of *pcDNA3.1-HA-Rybp* (i.e. 1 µg, 2.5 µg, 5 µg and 10 µg) was transfected by the CaPO_4_ method. Forty-eight hours after transfections, the cells were lysed using 1x cell culture lysis buffer (Cell culture lysis 5X reagent, Promega, E1531) and processed using the Dual Luciferase Reporter Assay System, (Dual Luciferase Reporter Assay System, Promega, cat. no. E1910) following the manufacturer's instructions. Twenty micrograms of the whole cell lysates from each sample was mixed with 100 µl of Luciferase Assay Reagent II (provided in the kit) and the luciferase activity was recorded immediately. The luciferase activity was recorded with Perkin Elmer TopCount NXT Luminometer. All measurements were taken in triplicates.

### 4.13. Western blot analysis

Expression analysis of proteins during *in vitro* cardiac differentiation was carried out by western blot technique. Whole cell lysates were isolated from differentiated samples by using 1x passive lysis buffer (5x Passive lysis buffer, Promega, cat. no. E1941). Concentration of the whole cell lysate was determined by the Bradford's method as mentioned above. The protein samples were stored in 6X Laemmli dye [59] and 20 µg of the quantified protein was then loaded in 10% sodium dodecyl sulfate–polyacrylamide gel electrophoresis (PAGE) using Bio-Rad Mini-Protean 3 cell, cat. no. 67S/11919. The protein was then transferred to polyvinylidene fluoride (transfer membrane, Immobilon-P, Millipore, cat. no. IPVH00010) and the membrane was hybed with anti-RYBP antibody (Anti-DEDAF, Merck Millipore, cat. no. AB3637, 1 : 1000) and anti-PLAGL1 antibody (Anti-Zac1 antibody (C-7), Santa Cruz, cat. no. sc-166944, 1 : 1000). Bio-Rad Goat-anti-mouse IgG-HRP conjugate, (cat. no. 172-101, 1 : 2000) and Merck Millipore Goat-anti-Rabbit IgG-HRP conjugate (cat. no. AP132P, 1 : 2000) were used as the secondary antibodies. The membranes were washed with TBST buffer (for five times with10 min of gentle shaking and then hybed with Immobilon Western, Chemiluminescent HRP Substrate, Millipore, cat. no. WBKLS0500. Alliance Q9 system (UVITECH) was used to capture the chemiluminescent signals.

### 4.14. Co-Immunoprecipitation

HEK293 cells were transiently transfected with 5 µg of *pcDNA3.1-Ring1a -FLAG, pRK7- FLAG* -*Nkx2*-5, *pRK7-FLAG-Mef2c* and *pRK7-FLAG-Plagl1* [60] in combination with 5 µg of *pcDNA3.1-Rybp.* Transient transfection and protein lysis were performed as mentioned above. The whole cell lysates were incubated in ice for 15 min and were spun at 15 000 rpm for 10 min at 4°C. The supernatant was separated and pre-cleaned with 30 µl of Protein A-Sepharose beads (Sigma, cat. no. P-3391) at 4°C under gentle rocking for 20 min. The precleared supernatant with Sepharose beads was spun at 2000 rpm for 2 min at 4°C. Eighty microlitres of the supernatant was collected and mixed with 6X Laemmli dye, boiled for 10 min at 100°C to use as input lysates for western blot analysis. The remainder of the supernatant was incubated overnight at 4°C under gentle rocking with 30 µl of RYBP antibody (Anti-DEDAF, Millipore, cat. no. AB3637) bound Sepharose beads (5 µl of RYBP antibody (1 µg ml^−1^) was bound to 100 µl of Sepharose beads for 4 h at 4°C under gentle rocking). To wash the immunoprecipitated proteins, the protein bound FLAG-tagged beads were centrifuged for at 2000 rpm for 2 min at 4°C and washed with 1X PBS for five times. The immunoprecipitated proteins bound to the FLAG-tagged beads were then mixed with 30 µl of 6X Laemmli dye, boiled for 10 min at 100°C and stored in −20°C until further use. Twenty microlitres of the input lysates and 20 µl of the immunoprecipitated proteins were loaded in 10% SDS-PAGE and western blot analysis (detailed in §4.13) was carried out. The western transferred membrane was immunoblotted with anti-FLAG antibody (Monoclonal Anti-FLAG M2 Peroxidase (HRP), Sigma, cat. no. A8592) at 4°C under gentle shaking overnight. The membranes were processed as described above.

### 4.15. Immunocytochemisty analysis

Immunofluorescence staining of *in vitro* cardiac cell cultures was achieved by culturing the cells over glass coverslips in 24-well plates (24-well Cell Culture Cluster Corning, cat. no. 3524) as described before and fixed with 4% para-formaldehyde (PFA, cat. no.) for 20 min in room temperature (RT). Cells were permeabilized by 0.2% Triton X-100 (Triton X-100, Sigma, cat. no. T8787) in PBS (Dulbeco PBS (1x), Gibco, cat. no. 14190-144) for 20 min in gentle shaking in RT. Five per cent BSA in PBS was used to block the cells for 1 h at RT. The cells were incubated with anti-RYBP antibody, (Anti-DEDAF, Merck Millipore, cat. no. AB3637) and anti-PLAGL1 antibody (Anti-Zac1 antibody (C-7), Santa Cruz, cat. no. sc-166944) both diluted in 5% BSA at 1 : 1000 dilution and incubated overnight in 4°C under gentle shaking. The cells were washed for five times with PBS and incubated with fluorescent labelled secondary antibody (Alexa Fluor 488 Goat-Anti-Rabbit, Invitrogen, cat. no. A-21206; Alexa Fluor 647 Donkey-Anti-Mouse, Invitrogen, cat. no. A-31571) at a concentration of 1 : 2000 in PBS for 1 h at 4°C. The cells were then washed three times with PBS. The cells were then incubated with 4′,6-diamidino-2-phenylindole (DAPI; Vector Laboratories, cat. no. H-1200) diluted in PBS at a concentration of 1 : 2500 for 20 min. The cells were then washed three times with PBS and mounted in Fluoromount-G (eBioscience, cat. no. 00-4958-02). The images were taken in Olympus LSM confocal microscopy (Olympus Corporation, Japan).

### 4.16. Analysis of reported expressed sequence tags of the Plagl1 splice variants

Complete CDS (coding sequence) of *Plagl1* mRNA and deposited transcript variants were downloaded in FASTA format from NCBI-Nucleotide database. Each variant sequence was BLASTed with the *Plagl1* genomic locus from Ensembl (https://www.ensembl.org/index.html) ID: ENSMUSG00000019817 as the reference file with indicating exon positions. The exons transcribed in each splice variant were identified and the splice variant sequences were aligned using BioEdit software. The corresponding position of the promoter region from which the splice variants were transcribed was presumed based on the coding exons and the relative position of the promoter regions.

### 4.17. Analysis of the Plagl1 promoter for CpG island and TATA box

The CpG islands in the *Plagl1 P1*, *P2* and *P3* promoters were analysed by uploading the FASTA sequence in the DBCAT online tool (http://dbcat.cgm.ntu.edu.tw). DBCAT uses methylation microarray data to analytically identify the CpG islands in the query sequence.

TATA box prediction was done by uploading the FASTA sequence of *Plagl1 P1*, *P2* and *P3* promoters into YAPP Eukaryotic core promoter prediction webtool (http://www.bioinformatics.org/yapp/cgi-bin/yapp.cgi).

### 4.18. Transcription factor binding analysis in Plagl1 promoters

Transcription factor binding (TFB) analysis was performed using TRANSFAC webtool (https://genexplain.com/transfac/). TRANSFAC is a widely used TFB analysis tool which identifies TFB sites based on the experimentally proven consensus of several TFs and ChIP binding [61–63]. The amplified and cloned *Plagl1* promoters *P1*, *P2* and *P3* promoter sequences were analysed for TFB sites by choosing either muscle specific, cell cycle specific and all eukaryotic TFs.

### 4.19. Metadata analysis in embryonic stem cells and cardiomyocytes

Metadata analysis for existing ChIP-seq analysis was performed by downloading pre-existing ChIP-seq data from GEO database (https://www.ncbi.nlm.nih.gov/geo/) under the following IDs. In ES cells, RYBP ChIP- GSM4052120, RNF2 ChIP- GSM4052131 [64] and input ChIP- GSM4052104, In cardiac progenitor cells, RYBP ChIP- GSM1657391, RNF2 ChIP- GSM1657390 and input ChIP- GSM1657392 [6].

For comparing the histone modifications in the *Plagl1* genomic locus, pre-existing ChIP-seq data were downloaded from GEO database under the following IDs. In ES cells [65]: H3K27me3—GSM1180182, H3K4me3—GSM1180179, H3K4me1—GSM1180178, H3K27ac1—GSM1180181 and input—GSM1180184. In cardiac progenitor cells [66]: H3K27me3—GSM1692788, H3K4me3—GSM1692789, H3K9ac1—GSM1692786, H3K27ac1—GSM1692787 and input—GSM1692806. The downloaded BigWig files were uploaded into IGV (Integrative Genomics Viewer) choosing specific annotations i.e. mm9 or mm10 according to the original analysis and the binding peaks were visualized by setting the data range of the peaks using the input file as the reference.

### 4.20. Statistical analysis

All experiments were repeated three times. Experiments were evaluated by using *t*-test type 3 for significance. All data are expressed as mean ± s.d. Values of *p* < 0.05; **p < 0.01; ***p < 0.001).

## Data Availability

The data are provided in the electronic supplementary material [67].
